# Nocturnal dipping profile in chronic kidney disease: Searching for underlying mechanisms in order to prevent adverse events

**DOI:** 10.14814/phy2.14665

**Published:** 2020-12-23

**Authors:** Marieta Theodorakopoulou, Konstantina Dipla, Andreas Zafeiridis, Pantelis Sarafidis

**Affiliations:** ^1^ Department of Nephrology Hippokration Hospital Aristotle University of Thessaloniki Thessaloniki Greece; ^2^ Laboratory of Exercise Physiology and Biochemistry Department of Physical Education and Sports Science at Serres Aristotle University of Thessaloniki Thessaloniki Greece

To the Editor,

We read with interest the study from Jeong et al. (2020), that investigated the potential role of sympathetic nervous system (SNS) overactivity and endothelial dysfunction in abnormal nocturnal blood pressure (BP) regulation in patients with chronic kidney disease (CKD). Hypertension is the most common modifiable risk factor in CKD, and is strongly associated with adverse outcomes (Sarafidis et al., 2017). Abnormal dipping pattern of nocturnal BP is present in 50%–80% of patients with CKD depending on the underlying CKD stage (Sarafidis et al., 2018) and has been associated with target‐organ damage (i.e., proteinuria and left ventricular hypertrophy), increased risk of CKD progression, as well as cardiovascular and total mortality (Parati et al., 2016; Sarafidis et al., 2017). SNS overdrive, salt‐sensitivity, sleep disorders, and endothelial dysfunction are considered to be main pathogenetic mechanisms (Parati et al., 2016).

Jeong et al. (2020), examined 32 hypertensive patients with CKD stage 2 and 3 with office BP, 24‐hr ambulatory BP, muscle sympathetic nerve activity (MSNA) and endothelial function via flow‐mediated dilation (FMD). This study is an important addition to current literature, as, to our knowledge it is the first to reveal that nocturnal BP profiles are associated with elevated SNS activation and impaired vascular endothelial function in patients with CKD. However, it has several limitations that may limit its conclusions. First, the total population is rather small. Second, the studied cohort included middle‐aged males, with the majority of them (93.75%) being African Americans, while the average weight was 106 kilograms and body mass index 32.7 kg/m^2^. Black race and obesity are known factors associated with increased SNS activity (Landsberg & Krieger, 1989; Richardson et al., 2013). Thus, the results have limited generalizability to the general CKD population; to what extent they apply into older individuals (i.e., the majority of CKD patients), female, Caucasian, and lean individuals is unknown. Third, study patients were divided into two groups (dippers and non‐dippers). This simplified classification (rather related to the small study size), is evidently the main study limitation, as relevant guidelines on ambulatory BP monitoring, include for years four categories of BP profile based on the night/day ratio: risers (ratio ≥ 1.0); non‐dippers (0.9 < ratio ≤1.0); dippers (0.8 < ratio ≤0.9); and extreme dippers (ratio ≤ 0.8) (O’Brien et al., 2013). An exaggerated MSNA in reverse dippers with essential hypertension was previously noted (Grassi et al., 2008); the authors do not report any relevant values in the Results section, but only write four lines in the discussion to report that MSNA was indeed numerically higher in reverse dippers compared to non‐dippers and dippers. A similar analysis on FMD results is not included.

In addition to the above, almost all black and most obese hypertensive patients, as well as most CKD patients are salt‐sensitive and current evidence suggests that endothelial dysfunction and SNS overdrive are involved in the pathogenesis of this salt‐sensitivity (Richardson et al., 2013; Sarafidis, 2008). Thus, it would also be nice for authors to examine salt‐sensitivity and enter it as a covariate in the regression analyses. Finally, a relationship between morning BP surge (MBPS) and cardiovascular outcomes is long‐known (Bilo et al., 2018). SNS overactivation and endothelial dysfunction are considered to be among the main pathophysiological mechanisms of MBPS (Bilo et al., 2018). Including data about MBPS and the relationship with MSNA and FMD would have increased the strength of the study. Overall, this interesting pilot study should be followed by larger, better designed efforts, to increase our understanding of these associations in CKD patients.

## CONFLICTS OF INTEREST

None regarding this manuscript.

## References

[phy214665-bib-0001] Bilo, G. , Grillo, A. , Guida, V. , & Parati, G. (2018). Morning blood pressure surge: pathophysiology, clinical relevance and therapeutic aspects. Integrated Blood Press Control, 11, 47–56. 10.2147/IBPC.S130277 PMC5973439 29872338

[phy214665-bib-0002] Grassi, G. , Seravalle, G. , Quarti‐Trevano, F. , Dell’Oro, R. , Bombelli, M. , Cuspidi, C. , Facchetti, R. , Bolla, G. , & Mancia, G. (2008). Adrenergic, metabolic, and reflex abnormalities in reverse and extreme dipper hypertensives. Hypertension, 52(5), 925–931. 10.1161/HYPERTENSIONAHA.108.116368 18779438

[phy214665-bib-0003] Jeong, J. H. , Fonkoue, I. T. , Quyyumi, A. A. , DaCosta, D. , & Park, J. (2020). Nocturnal blood pressure is associated with sympathetic nerve activity in patients with chronic kidney disease. Physiological Reports, 8(20), e14602 10.14814/phy2.14602 33112490 PMC7592496

[phy214665-bib-0004] Landsberg, L. , & Krieger, D. R. (1989). Obesity, metabolism, and the sympathetic nervous system. American Journal of Hypertension, 2(3 Pt 2), 125S–132S. 10.1093/ajh/2.3.125S 2647103

[phy214665-bib-0005] O’Brien, E. , Parati, G. , Stergiou, G. , Asmar, R. , Beilin, L. , Bilo, G. , Clement, D. , de la Sierra, A. , de Leeuw, P. , Dolan, E. , Fagard, R. , Graves, J. , Head, G. A. , Imai, Y. , Kario, K. , Lurbe, E. , Mallion, J.‐M. , Mancia, G. , Mengden, T. , … Zhang, Y. (2013). European society of hypertension position paper on ambulatory blood pressure monitoring. Journal of Hypertension, 31(9), 1731–1768. 10.1097/HJH.0b013e328363e964 24029863

[phy214665-bib-0006] Parati, G. , Ochoa, J. E. , Bilo, G. , Agarwal, R. , Covic, A. , Dekker, F. W. , Fliser, D. , Heine, G. H. , Jager, K. J. , Gargani, L. , Kanbay, M. , Mallamaci, F. , Massy, Z. , Ortiz, A. , Picano, E. , Rossignol, P. , Sarafidis, P. , Sicari, R. , Vanholder, R. , … Zoccali, C. (2016). Hypertension in chronic kidney disease part 2: role of ambulatory and home blood pressure monitoring for assessing alterations in blood pressure variability and blood pressure profiles. Hypertension, 67(6), 1102–1110. 10.1161/HYPERTENSIONAHA.115.06896 27141057

[phy214665-bib-0007] Richardson, S. I. , Freedman, B. I. , Ellison, D. H. , & Rodriguez, C. J. (2013). Salt sensitivity: A review with a focus on non‐Hispanic blacks and Hispanics. Journal of the American Society of Hypertension, 7(2), 170–179. 10.1016/j.jash.2013.01.003 23428408 PMC4574876

[phy214665-bib-0008] Sarafidis, P. A. (2008). Obesity, insulin resistance and kidney disease risk: Insights into the relationship. Current Opinion in Nephrology and Hypertension, 17(5), 450–456. 10.1097/MNH.0b013e328305b994 18695384

[phy214665-bib-0009] Sarafidis, P. A. , Persu, A. , Agarwal, R. , Burnier, M. , de Leeuw, P. , Halimi, J.‐M. , Heine, G. H. , Jadoul, M. , Jarraya, F. , Kanbay, M. , Mallamaci, F. , Mark, P. B. , Ortiz, A. , Parati, G. , Pontremoli, R. , Rossignol, P. , Ruilope, L. , Van der Niepen, P. , … Zoccali, C. (2017). Hypertension in dialysis patients: A consensus document by the European Renal and Cardiovascular Medicine (EURECA‐m) working group of the European Renal Association‐European Dialysis and Transplant Association (ERA‐EDTA) and the Hypertension and the Kidney working group of the European Society of Hypertension (ESH). Nephrology, Dialysis, Transplantation, 32(4), 620–640. 10.1093/ndt/gfw433 28340239

[phy214665-bib-0010] Sarafidis, P. A. , Ruilope, L. M. , Loutradis, C. , Gorostidi, M. , de la Sierra, A. , de la Cruz, J. J. , Vinyoles, E. , Divisón‐Garrote, J. A. , Segura, J. , & Banegas, J. R. (2018). Blood pressure variability increases with advancing chronic kidney disease stage: A cross‐sectional analysis of 16 546 hypertensive patients. Journal of Hypertension, 36(5), 1076–1085. 10.1097/HJH.0000000000001670 29465710

